# Post-stroke effects of IC87201 on neurobehavioral function and brain injuries: A stereological study^☆^

**DOI:** 10.1016/j.ibneur.2024.11.012

**Published:** 2024-11-24

**Authors:** Maryam Mohammadian, Aminollah Bahaoddini, Mohammad Reza Namavar

**Affiliations:** a Department of Biology, College of Sciences, Shiraz University, Shiraz, Iran; b Histomorphometry and Stereology Research Center and Department of Anatomical Sciences, Shiraz University of Medical Sciences, Shiraz, Iran; c Clinic Neurology Research Center, Shiraz University of Medical Sciences, Shiraz, Iran

**Keywords:** IC87201, Stereology, Striatum, Stroke

## Abstract

**Objectives:**

Stroke is the second leading cause of global death and is characterized by excitotoxic neuronal death caused by NMDA (N-Methyl-D-Aspartate) receptor overactivation. The present study was conducted to investigate the therapeutic potential of IC87201, a novel small molecule interfering with the NMDA receptor intracellular signaling pathway, in reducing the extent of ischemic stroke-induced brain damage.

**Materials and Methods:**

Cerebral ischemia was induced by the middle cerebral artery occlusion (MCAO) method in 24 anesthetized adult male rats for one hour. The animals were randomized into sham, MCAO, MCAO+ DXM (Dextromethorphan hydrobromide monohydrate) as an NMDA antagonist, and MCAO+ IC87201 groups which in the last two groups, DXM (50 mg/kg) and IC87201 (10 mg/kg) were injected intraperitoneally after ischemia. The neurobehavioral scores were appraised for 7 days and after that, brain tissue was appropriately prepared to perform the stereological evaluations.

**Results:**

The administration of IC87201 significantly recovered post-ischemia damages, including neurobehavioral function, reduction of volume of the total hemisphere, cortex, and striatum in rat brain, and the percentage of infarcted areas. Additionally, in the striatum region, IC87201 caused an increase in the total number of neuronal and non-neuronal cells as well as a decrease in the total number of dead cells. Some of these parameters were improved by DXM, but in general, IC87201 outperformed that.

**Conclusions:**

IC87201 was successful in minimizing ischemia-induced damage, especially in the striatal region. In addition, IC87201, as a molecule that acts on the intracellular signaling cascade of the NMDA receptor, performed better than DXM, as an antagonist of this receptor.

## Highlights

• Ischemic stroke in the striatum destroys neurons and non-neuronal cells
• Ischemic stroke in the striatum impairs the motor and sensory functions.
• IC87201 remarkably improves post-stroke striatal injuries and behavioral deficits.
• IC87201 had more beneficial effects on stroke than DXM, an NMDA receptor antagonist.

## 1 Introduction

Stroke as the major cause of brain dysfunction and long-term disabilities worldwide places a large burden on social service resources in most countries (Wu and Tymianski, 2018). Sensory and motor impairments such as lack of coordination and partial paralysis, central facial paresis, language and speech, cognitive deficits, impaired vision, depression, and altered levels of consciousness are common disabilities following stroke (Teo and Slark, 2016, Barthels and Das, 2020, Selakovic et al., 2019). Stroke is categorized into ischemic and hemorrhagic types, and ischemic represents the most common of cases (87 %) (Broderick et al., 2007).

The ischemic injury appears to be due to some abnormalities such as excitatory neurotransmitters-induced excitotoxicity, intracellular Ca^2+^ overload, and reactive oxygen species (ROS) formation (Wu and Tymianski, 2018). In particular, high concentrations of glutamate through NMDA (N-Methyl-D-Aspartate) receptors play a key role in the induction of ischemic and post-ischemic neuronal damage (Yang et al., 2011). NMDA receptors are widely expressed in the striatum, which is highly vulnerable to transient cerebral ischemia (Gan et al., 2014). It has been demonstrated in many studies that antagonists of glutamate receptors reduce cerebral ischemic damage. Despite the benefits of using antagonists of NMDA receptors, they have failed to become a therapeutic strategy for ischemic stroke patients because they can cause severe side effects such as nausea, vomiting, and cardiovascular and psychomimetic effects in treated patients (Wu and Tymianski, 2018). Thus, targeting NMDA receptor downstream signaling pathways can be considered an alternative therapeutic approach. At excitatory synapses, the scaffolding protein PSD-95 binds to both NMDA receptors and neuronal nitric oxide synthase (nNOS), forming a complex known as PSD95/nNOS. The association of nNOS with PSD-95 and calcium influx mediated by NMDA receptors are required for nNOS activation (Luo et al., 2014). Both soluble and particulate forms of nNOS are present in the brain, and after ischemia, nNOS and PSD-95 solubility are noticeably reduced. The amount of the nNOS increases in the membrane while decreasing in the cytosol in cultured neurons treated with glutamate and glycine. The translocation of this enzyme may be crucial in cerebral ischemia due to the fact that nNOS is necessary for NMDA receptor-dependent neuronal death (Zhou et al., 2010).

In the last decade, several chemical components have been discovered that can interfere with the interaction between the PSD95/nNOS complex as an intracellular multi-protein complex attached to NMDA receptors and, as a consequence, prevent nitrogenic free radical formation. IC87201 is a small molecule inhibitor of the PSD95-nNOS interaction, first introduced by Florio and colleagues in 2009 (Florio et al., 2009a). According to the literature, IC87201 prevents the interaction of PSD-95 and nNOS protein in vitro (Lee et al., 2015a) and attenuates glutamate-induced increases in NO production without affecting either the catalytic activity of nNOS (Zhou et al., 2010, Florio et al., 2009a) or NMDA receptor-mediated excitatory postsynaptic currents (Zhou et al., 2010).

Considering the IC87201 mechanism of action and its therapeutic potential in stroke, this study was designed to evaluate the effects of IC87201 on motor behavior impairment and striatum tissue damage induced by cerebral ischemia. Dextromethorphan hydrobromide monohydrate (DXM), an NMDA receptor antagonist, has additionally been used to compare the effects of blocking the NMDA receptor by modulating its activity through intracellular signaling pathways.

## 2 Materials and methods

### 2.1 Animals and drug administration

All procedures were performed based on the National Institutes of Health’s Guidelines for Care and Use of Laboratory Animals and Animal Research: Reporting in Vivo Experiments (ARRIVE) and approved by the local Ethics Committee of Shiraz University, Shiraz, Iran. The research work was approved by the Ethics Committee of the Biology Department, Shiraz University (SU-9531466). Adult Sprague-Dawley male rats, weighing 275–350 g were obtained from the Comparative and Experimental Medical Center of Shiraz University of Medical Sciences (SUMS), Shiraz, Iran. The rats were housed in the animal house with a controlled light cycle and temperature and available water and food ad libitum. Rats were acclimatized for one week prior to surgery. Twenty-four rats were randomly divided into four groups (n=6 per group): the sham, MCAO, MCAO+ Dextromethorphan hydrobromide monohydrate (DXM), and MCAO+ IC87201 groups. There was no drug administration or artery occlusion in the sham group. In the other three groups, transient brain ischemia was induced by the middle cerebral artery occlusion (MCAO) technique. Thereafter (4 hours after brain ischemia), normal saline, DXM (50 mg/kg) (Bokesch et al., 1994a), and IC87201 (10 mg/kg) (Smith et al., 2016) were intraperitoneally injected (as a single dose), respectively. The animals were evaluated by neurobehavioral function test 2 hours after surgery and every day for 7 days. After completing this period, animal brains were fixed, serially, and coronally sectioned and stained for histological and stereological studies.

### 2.2 Middle cerebral artery occlusion procedure

Transient brain ischemia was induced using the intraluminal filament technique described by Koizumi. They were anesthetized with intraperitoneal (i.p.) administration of Ketamine (100 mg/Kg) and Xylazine (10 mg/Kg). The right common carotid artery (CCA) was exposed through a midline incision on the neck and then carefully dissected from the vagus nerve and its sheath, external carotid artery (ECA), and internal carotid artery (ICA). A heat-rounded head and silicon-coated 4–0 monofilament nylon suture was inserted into the common carotid artery and advanced into the lumen of the ICA until it blocked the origin of the middle cerebral artery (MCA). One hour after MCAO, reperfusion was performed by withdrawing the suture. Finally, the incision was sutured and sprayed with oxytetracycline. The core body temperature was maintained at 37 ± 0.8 °C using a rectal thermometer and a heating pad throughout and after the surgery (Owjfard et al., 2020).

### 2.3 Neurological scores examination

Each rat was subjected to neurobehavioral evaluation 2 hours after surgery and daily for up to 7 days with the Garcia neurological test, assessing motor, sensory, reflex, and balance ability (Garcia et al., 1995). In this test, the highest and lowest possible scores are 18 and 6, respectively, indicating either no or severe cerebral damage. If the animal score was 18 or died from brain ischemia, they were excluded from the study and were replaced with another one.

### 2.4 Tissue processing

At the end of the 7th day after surgery, six rats in each group were deeply anesthetized with intraperitoneal administration of Ketamine and Xylazine and then, transcardially perfused through the left ventricle with 0. 9 % saline, followed by 4 % buffered paraformaldehyde. Brains were removed and stored overnight in the same fixative. Dissected brains were then embedded in paraffin and serially sectioned (25 µm thickness). These sections were selected for cresyl violet staining using a systematic random sampling protocol (Ebrahimian et al., 2017). Every 17th section (at an interval of 425 µm) was mounted on the slide and stained by cresyl violet. Finally, the stained slides were evaluated using a light microscope.

### 2.5 Stereological assessments

The total and infarcted volume of the ischemic hemisphere, cortex, and striatum were unbiasedly estimated by means of an unbiased estimator of volume based on Cavalieri’s principle using stereology software (StereoLite, SUMS, Shiraz, Iran). The brain areas were selected based on the rat brain stereotaxic atlas (Paxinos and Watson, 2006). The ischemic volume of the hemisphere, cortex, and striatum (V_ref_) was determined by applying the following formula: *V*_*ref*_ *= d×a(p)×∑P*, where, *d* = 425 µm; equals to the section interval or distance from one section to the next, *a(p)* = 191,844 µm^2^; equal to the area associated with one point in the grid to the next, and *∑P* is the number of points separately hitting any part of the specified structure (hemisphere, cortex and striatum and ischemic areas in them) projected on the test grid (Owjfard et al., 2020).

Furthermore, the total number of neurons, non-neurons, and dead neurons in the right striatum was counted in different groups, using the optical disector technique, (Mouton, 2002; Namavar et al., 2012). Briefly, the optical disector setting consisting of an Eclipse microscope (E200, Nikon, Tokyo, Japan) with a high-numerical-aperture (NA = 1.4) × 60 oil-immersion objective, was connected to a video camera. It transmits the microscopic image to a monitor, and an electronic microcator with digital readout (MT12, Heidenhain, Traunreut, Germany) for measuring the movements in the Z-direction with 0.5-μm precision. A computer-generated counting frame was superimposed on the screen, using a stereology software system (StereoLite, SUMS, Shiraz, Iran). The neuronal, non-neuronal, and dead neuronal numerical density is defined as follows: *Nv= ∑Q/[∑P×a(f)×h]*. where *∑Q* is the number of neurons, non-neurons, or dead neurons counted within the sampling frame or disector, *∑P* is the number of disector, *a(f)* = 1174 µm^2^, is the area of the sampling frame, and *h* = 15 µm, is the height of the sampling frame in the Z-direction (Namavar et al., 2020, Namavar et al., 2012).

The accuracy of the estimates was expressed by the coefficient of error (CE). We calculated the CE of our estimation, utilizing the method by Gundersen et al. (1999). An average of 12 (±0.5) sections was counted per brain. A sufficient amount of about 4421 (±206) points per hemisphere was counted on all areas giving the mean CE on the estimates of the total volume of 4 %. Neuronal nuclei were counted in approximately 680 (±46) optical disectors, 2171 (±168) neurons (CE=7 %), 3106 (±195) non-neurons (CE=6 %), and 259 (±33) dead neurons (CE=12 %) per striatum in each animal.

Neurons and non-neurons are two different types of brain cells. A normal neuron has the following basic morphology when stained with cresyl violet: a large cell body or perikaryon with neurites projecting from the cell body, Nissl substance in the perikaryon, and a recognizable nucleus that is always pale or euchromatic with a distinct nucleolus. Dead neurons consist of apoptotic or necrotic cells that exhibit an accumulation of dense globular material in the cytoplasm along with signs of nuclear fragmentation, shrunken perikarya, and smaller, darkly stained nuclei (Namavar et al., 2020). Also, the percentage of infarcted volume area for the ischemic hemisphere, cortex, and striatum was calculated through this formula:)Ischemic Infarct Volume/Total Volume(×100

### 2.6 Statistical analysis

All data were analyzed by Graph Pad Prism software (version 9. 1. 0.), and expressed as mean ± SEM. Statistical differences were measured by one-way analysis of variance (ANOVA) followed by the Tukey post hoc test for multiple group comparisons. Repeated measure two-way ANOVA followed by the Tukey post hoc test was used for statistical evaluation of the behavioral scores of the Garcia neurological test. Values of P<0.05 were considered significant.

## 3 Results

### 3.1 Neurobehavioral function

**Fig. 1:**
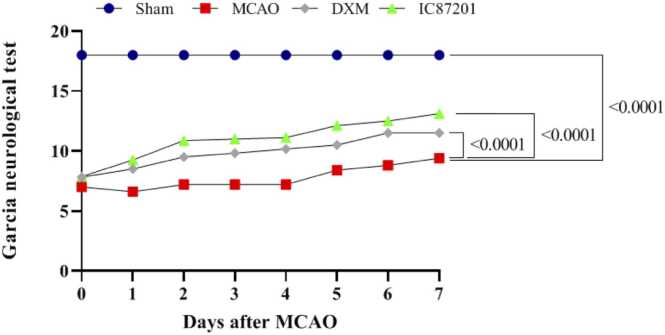
Evaluation of neurobehavioral function improvement using Garcia neurological test in, MCAO, MCAO+ DXM, and MCAO+IC87201groups 2 hours after surgery (day 0) and daily up to 7 days. (Data are presented as mean ± SEM and analyzed using a two-way ANOVA followed by Tukey post hoc test, n=6).

As previously mentioned, the neurobehavioral deficits score was determined by the Garcia neurological test. As shown in Fig. 1, the mean of neurobehavioral scores in the MCAO, MCAO+DXM, and MCAO+IC87201 groups at 2 h after brain ischemia (day 0) were 7, 7.83, and 7.87, respectively. Considering Garcia's test scoring system, these low scores indicate that ischemia was successfully induced. The neurobehavioral scores significantly increased from the second postoperative day in the groups administered with DXM and IC87201 compared with the MCAO group, indicating an improving trend in the presence of these two medications. Additionally, DXM and IC87201 performed nearly identically when compared in terms of ameliorating the neurobehavioral damage.

### 3.2 Stereological assessment

**Fig. 2:**
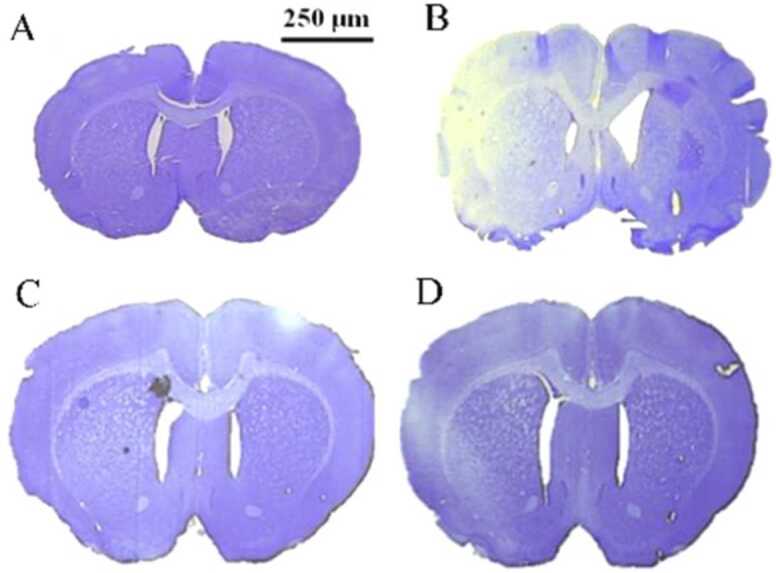
Cresyl violet stained photo of brain tissue sections in different groups: (A) Sham, (B) MCAO, (C) MCAO+ DXM, and (D) MCAO+ IC87201. The non-ischemic region of the brain shows up as a dark blue to purple color (A), whereas the ischemic areas appear very pale blue to white (B, C, and D).

In cresyl violet stained sections, the non-ischemic region of the brain shows up as a dark blue to purple color (Fig. 2A), whereas the ischemic areas appear very pale blue to white (Fig. 2, B, C, and D)

**Fig. 3:**
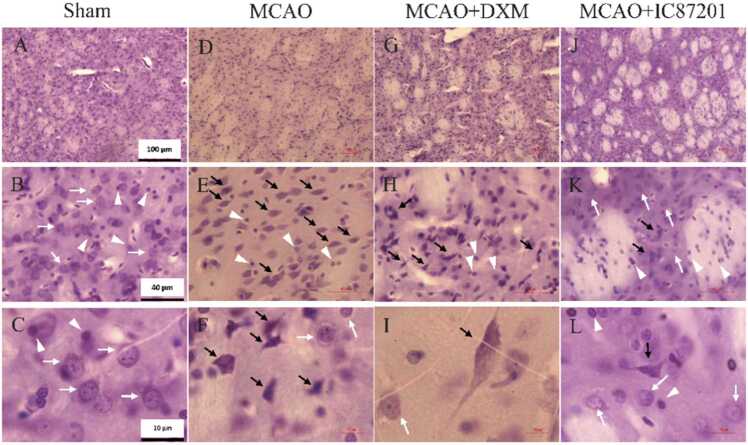
Representative Cresyl Violet-stained photograph of the brain tissue sections in different groups: (A, B, and C) Sham group with normal neurons (white arrow) and non-neuron cells (white arrowhead); (D, E, and F) Rats were subjected to MCAO showed frequent dead cells (black arrow); (G, H, I) MCAO+DXM and (L, K, L) MCAO+ IC87201 groups showed fewer dead neurons (black arrow).

Right striatum histological evaluation at the microscopic level in the sham group showed normal neural tissue with normal neurons having recognizable nucleolus and euchromatic nucleus as well as Nissl-stained cytoplasm without dead cells (Fig. 3A-C). While in the MCAO group, numerous dead neurons with shrunken nuclei and perikarya were observed (Fig. 3D-F). These dead cells were considerably diminished in the presence of IC87201 (Fig. 3J-L). The group receiving DXM, however, did not reveal this reduction (Fig. 3G-I).

**Fig. 4:**
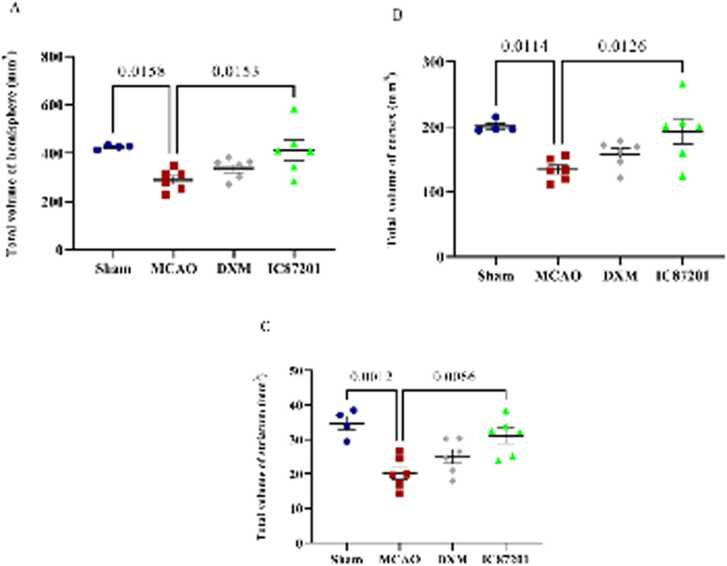
The total volume of the hemisphere (A), cortex (B), and striatum (C) in Sham (n=4), MCAO, MCAO+ DXM, and MCAO+ IC87201groups (n=6). (Data are presented as mean ± SEM and analyzed using one-way ANOVA followed by Tukey post hoc test for multiple group comparisons).

The volume of the total right hemisphere, cortex, and striatum was significantly lower after middle cerebral artery occlusion than it was in the sham group. Despite the fact that DXM administration increased hemispheric, cortical, and striatal volumes, this effect was not statistically significant. In the group receiving IC87201, these volumes did not decrease significantly, so it was comparable to the sham group (Fig. 4A-C).

**Fig. 5:**
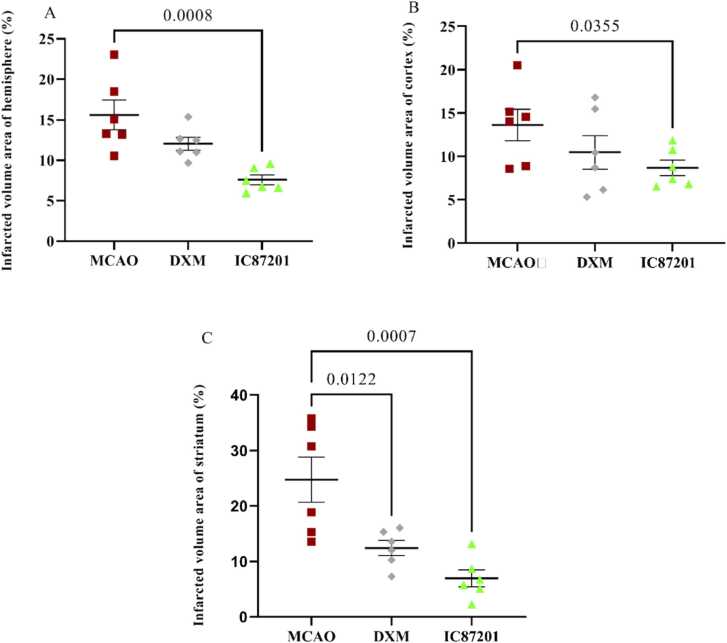
The volume percentage of infarcted area in the hemisphere (A), cortex (B), and striatum (C) in MCAO, MCAO+ DXM, and MCAO+ IC87201 groups. (Data are presented as mean ± SEM and analyzed using one-way ANOVA, followed by post hoc analyses using the Tukey test for multiple comparisons, n=6).

As mentioned previously, the percentage of infarcted volume was separately calculated for the ischemic hemisphere, cortex, and striatum. Considering the comparison of infarcted percentages in different regions of the brain, the striatum had the highest percentage of ischemic volume (24.76±4.07), while the hemisphere (15.87±1.83) and the cortex (13.55±1.81) showed relatively lower levels of ischemia. This study also showed that the administration of IC87201 significantly reduces the percentage of the infarct area in all three parts of the brain, while DXM improved this parameter only in the striatum (Fig. 5A-C).

**Fig. 6:**
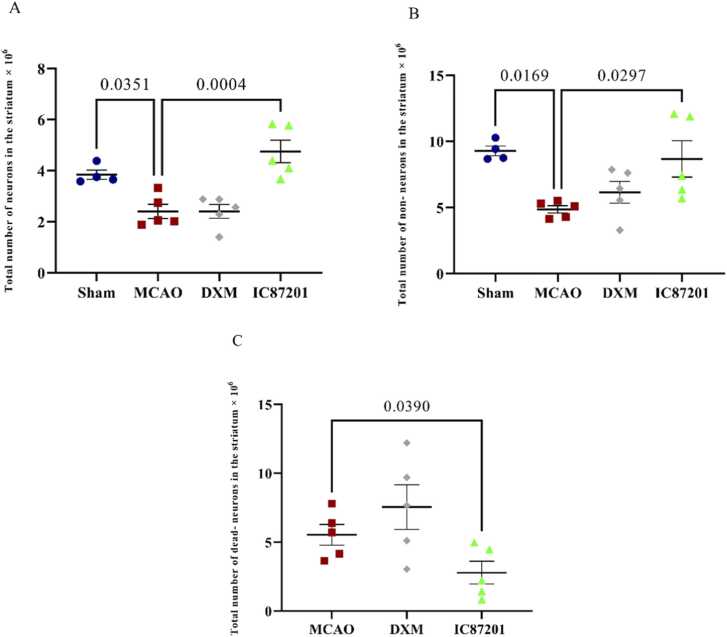
The striatum total number of neurons (A), non-neurons (B), and dead- neurons (C) in the Sham (n=4), MCAO, MCAO+ DXM, and MCAO+ IC87201 groups (n=5). (Data are presented as mean ± SEM and analyzed using one-way ANOVA, followed by Tukey post hoc test for multiple group comparisons).

Comparing the MCAO to the sham group, a significant decrease in the total number of neurons in the striatum was observed. As shown in Fig. 6A, a reduction in the number of neurons also occurred in the presence of DXM, but treatment with IC87201 prevented this neuronal loss. Following cerebral ischemia, the striatal total number of non-neurons was remarkably decreased. This parameter was recovered only in the IC87201 injected group (Fig. 6B). Brain ischemia in the MCAO group led to a significant number of dead neurons. The number of dead neurons did not decrease despite the administration of DXM. However, this parameter considerably dropped after the injection of IC87201 compared with the MCAO group (Fig. 6C).

## 4 Discussion

This is the first report investigating the effects of a new synthetic molecule, IC87201, on post-stroke neurobehavioral function and tissue injury in the striatal area through stereological evaluation. The middle cerebral artery occlusion (MCAO) model described by Koizumi is a valuable and reproducible animal model for simulating human ischemic stroke (Fluri et al., 2015), and we adopted this suitable model in our investigation.

In the current study, the MCAO significantly decreased the neurobehavioral score as determined by the neurological Garcia test. Additionally, stereological analysis of the ischemic hemisphere, cortex, and striatum revealed a reduction in the volume and number of neurons and non-neurons as well as a creation of a significant number of dead cells, which could be the explanation for the behavioral outcomes. In the presence of IC87201, these damaged parameters caused by cerebral ischemia recovered to almost normal levels.

According to the literature, neurodegeneration in the striatum frequently happens following middle cerebral artery occlusion (Popp et al., 2009, Radenovic et al., 2011). The striatum, as the primary part of the basal ganglia, expresses a high density of NMDA receptors and is supplied with numerous glutamatergic pathways originating from the sensorimotor cortex and the subthalamic nucleus. These receptors are essential for cognition and motor coordination, as well as being involved in the pathogenesis of neurodegenerative diseases (Gan et al., 2014, Radenovic et al., 2011). During the ischemic period and early reperfusion, excessive release of excitatory amino acids, intracellular overload of Ca^2+^, and increased free radicals are characterized as hallmarks of excitotoxicity. Therefore, glutamate release and NMDA channel activation are one of the main causes of ischemia-induced cellular injuries. The neuroprotective efficiency of NMDA receptor antagonists is widely studied in research. In the adult gerbil striatum and hippocampus, post-ischemic treatment with MK-801 as a non-competitive NMDA receptor antagonist provided significant neuroprotection against global brain ischemia and improved neurological status (Radenovic et al., 2011). Furthermore, the neuroprotective effects of dextromethorphan, as another non-competitive NMDA receptor antagonist, have been investigated and confirmed in numerous post-cerebral ischemia studies (Werling et al., 2007). Although previous research has highlighted the therapeutic potential of dextromethorphan (DXM) in mitigating ischemic injury, our findings did not demonstrate a statistically significant neuroprotective effect of DXM. This discrepancy may stem from the dose-dependent properties of DXM.

In this study, the selected dose of DXM was informed by earlier investigations reporting its neuroprotective effects (Bokesch et al., 1994b, Schmid-Elsaesser et al., 1998, Yin and Sun, 1999). However, it is important to recognize that the effectiveness of DXM is highly dependent on dosage, with excessive doses linked to detrimental effects. High-dose DXM has been associated with adverse outcomes such as seizures, neuronal death, suppression of anti-apoptotic factors (e.g., Bcl-2 and Bcl-Xl), upregulation of pro-apoptotic markers like Bax and caspase-3, and damage to vital brain structures (Tran et al., 2017). The lack of significant neuroprotection observed in our study may be due to the toxic side effects associated with higher doses of DXM, which could have counteracted its potential therapeutic effects. Although the chosen dosage was based on previous studies, the possibility remains that the toxicity at this dose impaired the drug's efficacy in our specific experimental conditions.

Despite the positive effects of NMDA receptor antagonists in some pre-clinical studies, these compounds have serious side effects in treated patients, including nausea, vomiting, cardiovascular problems, and psychomimetic effects. Additionally, NMDA receptors play a dual role in the neuron`s survival and death, so that, the stimulation of synaptic and extrasynaptic NMDA receptors activates pro-survival and pro-death pathways, respectively (Wu and Tymianski, 2018). As a result, the useful function of NMDA receptors is eliminated when their antagonists are used. Therefore, it appears that NMDA receptor blockade, even at doses that may be therapeutic, interferes with normal neuronal function and results in significant side effects.

Due to the lack of clinical success with NMDA receptor antagonists, the focus of stroke neuroprotection shifted towards the identification of downstream intracellular signaling pathways triggered by NMDA receptors. IC87201, as a de novo small molecule, was synthesized to selectively dissociate the GluN2B-PSD95-nNOS complex in the intracellular side of the NMDA receptor, without inhibiting the normal nNOS activity in neurons (Florio et al., 2009a). The association of postsynaptic density protein 95 (PSD95) with neuronal nitric oxide synthase (nNOS) plays an important role in stroke-induced neuronal damage. The nNOS/PSD95/NMDA receptor ternary complex causes local production of NO as a result of glutamate-mediated Ca^2+^ influx through the NMDA receptor. In some pathological conditions related to glutamate excitotoxicity such as ischemic stroke, neuropathic pain, and neurological diseases, overactivation of the NMDA receptor leads to excessive production of NO and consequently neuronal death (Bach et al., 2015). Some new synthetic compounds, such as IC87201, which are proposed as nNOS/PSD95/NMDA receptor inhibitors, can target the PDZ (PSD95/Disc large/Zonula occludens) domains of PSD95 and thus prevent the harmful production of NO as a result of NMDA receptor overactivation. In 2009, Florio et al. showed the ability of IC87201 to prevent the interaction between nNOS and PSD95 with an IC50 of 31 µM (Florio et al., 2009b). Also, in another study, the potential of IC87201 in disrupting the interaction between PSD-95 (1−392) and nNOS (1−199) was confirmed with an EC50 of 23.9 μM, using the fluorescent-based in vitro assay AlphaScreen (Lee et al., 2015b). However, there are also controversial results in this field. In a biochemical study, it was shown that although the use of IC87201 in neurological and animal models of ischemic stroke, pain, and depression seems very convincing, IC87201 does not bind the extended nNOS-PDZ domain (or the PSD95-PDZ domains) and is not able to inhibit nNOS-PDZ/PSD95-PDZ interactions (Bach et al., 2015). Therefore, it is possible that IC87201 exerts its effect by binding to other parts of the large nNOS protein, or by interacting with other proteins affecting the nNOS/PSD95 system. Certainly, more extensive studies are needed to understand the precise mechanism of action of IC87201.

IC87201 has been studied for its antidepressant effect (Doucet et al., 2015), improving source and spatial memory, and motor function (Smith et al., 2016), and reducing NMDA-induced hyperalgesia in mice (Lee et al., 2015a). However, there are limited reports on its protective effects following cerebral ischemia. In our previous studies, we demonstrated that IC87201 mitigates post-stroke injuries by restoring total and infarcted volumes, preserving the total number of neurons and non-neurons, and reducing neuronal death in the CA1 and CA3 regions of the hippocampus in rats. Additionally, it ameliorated ischemia-induced learning and memory impairments, with behavioral improvement correlating significantly with structural recovery in the hippocampus (Mohammadian et al., 2024). Furthermore, we reported that IC87201 reduces cardiac complications of cerebral ischemia by suppressing sympathetic overactivation and improving heart rate variability (HRV) parameters (Bahaoddini, 2023).

Recent in vitro research also supports the neuroprotective potential of IC87201. In cultured hippocampal neurons, IC87201 reduced cGMP levels, a marker of nitric oxide (NO) production triggered by NMDA stimulation (Smith et al., 2016). Furthermore, in cultured cortical neurons, IC87201 dose-dependently attenuated neuronal injury and apoptotic cell death (Gan et al., 2014). Studies on ZL006, a structural homolog of IC87201, have similarly demonstrated neuroprotective properties, including reduced ischemic brain damage in mouse and rat stroke models (Zhou et al., 2010). These findings align with and reinforce our current results, highlighting the therapeutic potential of IC87201 in ischemia-related neuronal damage.

In addition to damaging neurons, glial cells are also destroyed by ischemia. Brain edema due to ischemic events in rats lasts up to 24 hours and after that edema cannot affect the volume of the structure (Bach et al., 2015). We evaluated the volume 7 days after brain ischemia. Therefore, this phenomenon will not affect the volume of the brain. Cerebral ischemia can lead some glial cells, such as microglia, to develop a pro-inflammatory profile releasing cytokine-like interleukin-1 or TNF-α, which can polarize astrocytes toward a neurotoxic phenotype and exacerbate the inflammatory response (Hernández et al., 2021). The NMDA receptors are expressed by a subset of glial cells, including oligodendrocytes and astrocytes. These cells are damaged by excessive activation of NMDA receptors following ischemia due to intracellular calcium overload (Dzamba et al., 2013). Based on the expression of NMDA receptors in glial cells, it is possible that IC87201 prevents the death of these cells by the same mechanism as it acts on neurons, i.e. reducing excitotoxicity. However, it should be noted that in this study, non-neuronal cells including microglia, oligodendrocytes, astrocytes, and vascular endothelial cells have generally been investigated due to their potential to have either positive or negative effects in ischemic conditions. Therefore, to confirm the accuracy of our claim, a growing body of specialized research is required. Also, due to the lack of differential techniques, non-neuronal terms including glial and endothelial cells have been used instead of glial cells in this study.

The striatum showed the highest percentage of ischemia according to the current finding, suggesting that the striatum was likely inside the ischemic core. These observations may be explained by the finding that cerebral blood flow (CBF) in cortical branches returns to normal levels within 120 min of reperfusion, but striatal CBF values remain severely ischemic after longer reperfusion (Owjfard et al., 2020). As a result, the striatum serves as an ischemic core after the occlusion of the middle cerebral artery, and this study supports earlier research that found the cortex and striatum are the main brain regions affected by cerebral ischemia (Carmichael, 2005).

In terms of IC87201 effects on improving cerebral ischemia, there are no similar studies to compare with our results. However, it has been demonstrated that ZL006, a synthetic substance with a very similar structure and function to IC87201, has neuroprotective effects in vitro and ameliorates cerebral ischemic injury in mouse and rat stroke models (Zhou et al., 2010).

The current study has some limitations which have to be pointed out. For example, the changes observed in the number of glial cells in this study should be investigated using immunohistochemical techniques to assess the diversity of glial cell types as well as the ability of these cells to switch between deleterious and protective phenotypes in the presence of IC87201. Also, to confirm our findings about the therapeutic effect of IC87201, further molecular studies on the nNOS enzyme's activity and the number of free nitrogen radicals in the presence of this agent are necessary.

### 4.1 Conclusion

Despite our limitations, the findings of our research demonstrate that IC87201 has been successful in minimizing the striatal neuronal and non-neuronal damage, infarcted volume, and neurobehavioral deficits induced by cerebral ischemia. These effects could be attributed to IC87201's potential to reduce ischemia-induced excitotoxicity. Additionally, IC87201 outperformed DXM, an NMDA receptor antagonist, in treating the aforementioned injuries. Regarding the novelty of IC87201, more extensive research is required to fully investigate its therapeutic potential in post-ischemic conditions.

## Ethics statements

All procedures were performed based on the National Institutes of Health’s Guidelines for Care and Use of Laboratory Animals and Animal Research: Reporting in Vivo Experiments (ARRIVE) and approved by the local Ethical Committee of Shiraz University (SU-9531466).

## Ethics Statement

The protocol for the research project has been approved by the Ethics Committee of Shiraz University (Ethical code: SU-9531466) and was conducted following the National Institutes of Health’s Guide for Care and Use of Laboratory Animals and the Animal Research: Reporting *In Vivo* Experiments (ARRIVE) Guidelines.

## Grant support

This work was supported by a grant from Shiraz University, Shiraz, Iran (Grant number: SU9531466), which was extracted from Maryam Mohammadian's Ph.D. thesis.

## CRediT authorship contribution statement

**Maryam Mohammadian:** Writing – original draft, Software, Project administration, Methodology, Investigation. **Aminollah Bahaoddini:** Writing – review & editing, Validation, Resources, Funding acquisition, Conceptualization. **Mohammad Reza Namavar:** Writing – review & editing, Visualization, Validation, Supervision, Project administration, Conceptualization.

## Declaration of Generative AI and AI-assisted technologies in the writing process

We declare did not use any Generative artificial intelligence (AI) or AI-assisted tools in writing the manuscript or to create or alter images in submitted manuscripts.

## Declaration of Competing Interest

The authors declare that they have no conflict of interest. We declare did not use any Generative artificial intelligence (AI) or AI-assisted tools in writing the manuscript or to create or alter images in submitted manuscripts.

## Data Availability

The data that support the findings of this study are available on request from the corresponding author. The data are not publicly available due to privacy or ethical restrictions.
